# Viscoelasticity of periodontal ligament: an analytical model

**DOI:** 10.1186/s40759-015-0007-0

**Published:** 2015-11-16

**Authors:** Sergei M. Bosiakov, Anna A. Koroleva, Sergei V. Rogosin, Vadim V. Silberschmidt

**Affiliations:** Department of Mechanics and Mathematics, Belarusian State University, 4, Nezavisimosti Avenue, Minsk, 220030 Belarus; Department of Economics, Belarusian State University, 31, K. Marx, Minsk, 22030 Belarus; Institute of Mathematics, Physics and Computer Science, Department of Mathematics, Aberystwyth University, Penglais, Aberystwyth Ceredigion, SY23 3BZ UK; Wolfson School of Mechanical and Manufacturing Engineering, Loughborough University, Leicestershire, LE11 3TU UK

**Keywords:** Periodontal ligament, Tooth root, Viscoelastic model, Fractional exponential function, Translational displacement

## Abstract

**Background:**

Understanding of viscoelastic behaviour of a periodontal membrane under physiological conditions is important for many orthodontic problems. A new analytic model of a nearly incompressible viscoelastic periodontal ligament is suggested, employing symmetrical paraboloids to describe its internal and external surfaces.

**Methods:**

In the model, a tooth root is assumed to be a rigid body, with perfect bonding between its external surface and an internal surface of the ligament. An assumption of almost incompressible material is used to formulate kinematic relationships for a periodontal ligament; a viscoelastic constitutive equation with a fractional exponential kernel is suggested for its description.

**Results:**

Translational and rotational equations of motion are derived for ligament’s points and special cases of translational displacements of the tooth root are analysed. Material parameters of the fractional viscoelastic function are assessed on the basis of experimental data for response of the periodontal ligament to tooth translation. A character of distribution of hydrostatic stresses in the ligament caused by vertical and horizontal translations of the tooth root is defined.

**Conclusions:**

The proposed model allows generalization of the known analytical models of the viscoelastic periodontal ligament by introduction of instantaneous and relaxed elastic moduli, as well as the fractional parameter. The latter makes it possible to take into account different behaviours of the periodontal tissue under short- and long-term loads. The obtained results can be used to determine loads required for orthodontic tooth movements corresponding to optimal stresses, as well as to simulate bone remodelling on the basis of changes in stresses and strains in the periodontal ligament caused by such movements.

## Background

A root of a tooth is attached to an alveolar bone by a periodontal ligament (PDL), a soft connective tissue consisting of collagen fibres and a matrix phase with nerve endings and blood vessels (Bergomi et al. 2011; Berkovitz et al. 1995; Chatterjee 2006; Chiba 2004; Fill et al. 2011; Nanci and Ten Cate AR 2008; Natali 2003; Nishihira et al. 2003). In addition to providing interconnection for a tooth with its supporting structures, the PDL responds to applied loads, demonstrating viscoelastic time-dependent properties (Burstone et al. 1978; Jonsdottir et al. 2006; Komatsu 2010; Komatsu et al. 2007; Middleton et al. 1996; Picton 1990; Qian et al. 2009; Ross et al. 1976; Toms and Eberhardt 2003).

Depending on duration of the applied load, initial and orthodontic tooth motions are distinguished (Dorow et al. 2003; Frost 1992; Kawarizadeh et al. 2003; Middleton et al. 1996). The former occurs under a short-term load, with the tooth returning to its original position after load removal (Muhlemann and Zander 1954; Tanne et al. 1991; Ziegler et al. 2005), accompanied by a rearrangement of bone tissue. Thus, the PDL plays an important role in ensuring a proper reaction of bone. Analysis of biological mechanisms underpinning tooth movements (Davidovitch and Shanfeld 1975; Proffit et al. 1993; Reitan and Rygh 1994) showed that stresses and strains in the PDL, caused by external forces, were a key driver of bone reconstruction. Stretching and compression the PDL tissue lead to resorption and bone formation, respectively (Bourauel et al. 2000; Davidovitch et al. 1980; Masella and Meister 2006; Melsen 2001; Roberts and Chase 1981; Storey 1973; Wise and King 2008).

Short- and long-term (orthodontic) teeth motions can be modelled employing a linear elastic, bilinear elastic, viscoelastic, hyperelastic, or multiphase formulation for the PDL Bergomi et al. 2011; Cattaneo et al. 2005; Ferrari et al. 2008; Fill et al. 2012; Kawarizadeh et al. 2003; Middleton et al. 1996; Muraki et al. 2004; Natali et al. 2002; Natali et al. 2011; Provatidis 2000, Qian et al. 2009). The same type of continuous models are used to calculate stress-strain states of the PDL for various load types, as implemented in different finite-element studies (Cattaneo et al. 2005; Clement et al. 2004; Dorow and Sander 2005; Ferrari et al. 2008; Hohmann et al. 2011; Jeon et al. 1999; Jones et al. 2001; Kawarizadeh et al. 2003; Muraki et al. 2004; Natali et al. 2004; Pietrzak et al. 2002, Provatidis 2000; Qian et al. 2009; Reimann et al. 2009; Toms and Eberhardt 2003; Vollmer et al. 1999, Ziegler et al. 2005). Because of their complexity, analytical modelling of elastic and viscoelastic responses of PDLs to loads applied to the tooth was carried out in a relatively small number of studies (Kusy and Tulloch 1986; Natali et al. 2007; Nikolai 1996; Pena et al. 2007, 2008a,b; Provatidis 2001; Slomka et al. 2008; Smith and Burstone 1984; Van Schepdael et al. 2013). Most important results for 3-D cases were obtained using circular and elliptic-paraboloid shapes for the tooth root and PDL surfaces (Haack and Haft 1972; Provatidis 2001; Van Schepdael et al. 2013). In addition, results of a study by Bourauel et al. (2000) demonstrated that approximation of the actual geometry of the tooth with a paraboloid having an elliptical cross-section allows modelling the short-term and orthodontic tooth movements with high accuracy. In studies of Provatidis (2001), Van Schepdael et al. (2013), models a tooth root and a PDL in the form of a paraboloid were used to identify the magnitudes of initial displacements under static loads, stress-strain states of the PDL, a position of the centre of resistance of the tooth, as well as the effect of eccentricity of a cross section at initial displacements. An important feature of the used analytical model was an approximation of the PDL as an almost incompressible material with a Poisson’s ratio equal to 0.4–0.49 (Rees and Jacobsen 1997). In this case, it can be assumed that maximum deformation of the PDL tissue along a normal to the tooth-root surface coincides with thickness of the PDL in the same direction. Finite-element studies of the PDL’s stress-strain state under instantaneous loads (corresponding to small displacements of the tooth root) indicated high accuracy of the analytical model. A further development of the analytical scheme for the almost incompressible PDL proposed by Provatidis (2001) can be implemented for long-term and heavy loads, taking into account time-dependent and viscoelastic properties of the PDL.

The aim of our study is to develop an analytical model of a viscoelastic PDL with a fractional exponential kernel to describe evolution of deformation in a periodontal tissue and evaluate tooth-root movements with time. Viscoelastic behaviour of the periodontal ligament is in agreement with the widely employed Nutting law (Koeller 1984; 2010; Mainardi 2010; Uchaikin 2013) that can be simply presented in the form of the dependence of the shear stress using of strain and time. Such a relationship is suitable when the material properties are determined by various states between an elastic body and a viscous fluid.

## Methods

### Geometrical form of tooth root and PDL

In the suggested approach, an external surface of a tooth root (supposed to be a rigid body) and an adjacent inner surface of the PDL are modelled with a paraboloid (Van Schepdael et al. 2013)

(1) $$ F(x,y,z) = \frac{y}{h} - \frac{1}{b^{2}} \left((1-e^{2}) x^{2} + z^{2} \right) = 0,  $$

where *h* is the height of the tooth root; $e = \sqrt {1-(b/a)^{2}}$ is the eccentricity of the elliptical cross-section of the tooth in the alveolar crest; *a* and *b* are the semi-axes of this ellipse.

The internal surface of the PDL adjacent to the dental alveolar bone is shifted along the normal to the surface of the tooth root. Its equation is as follows:

$$\begin{aligned} F_{1}(x,y,z) &= \frac{y + n_{y} \delta}{h} - \frac{1}{b^{2}} \left((1-e^{2}) (x + n_{x} \delta)^{2}\right.\\ &\quad\left.+\, (z+n_{z}\delta)^{2}\right) = 0, \end{aligned} $$

where *n*_*x*_, *n*_*y*_, and *n*_*z*_ are the components of the unit normal vector to the surface of the first paraboloid; *δ*>0. The components of the normal vector are determined from (1):

(2) $$ \begin{aligned} n_{x} &= - \frac{2 (1-e^{2}) h x}{b^{2} \Delta}, n_{y} = \frac{1}{\Delta}, n_{z} = - \frac{2 h z}{b^{2} \Delta},\\ \Delta &= \frac{1}{b^{2}} \sqrt{b^{4} + 4 h^{2} ((1-e^{2})^{2} x^{2} + z^{2})}. \end{aligned}  $$

Under a concentrated force, points of the PDL on the tooth-root surface (1) begin to move with the root, while the external surface of the PDL is fixed. There is no significant difference between the schemes considering fixing of the outer surface of the PDL to the alveolar bone or its full constraint. Hence, to calculate the initial movement of the teeth in the PDL, both the teeth and the alveolar bone could be considered as solids (Hohmann et al. 2011).

### Expressions for strains and displacements

**Fig. 1:**
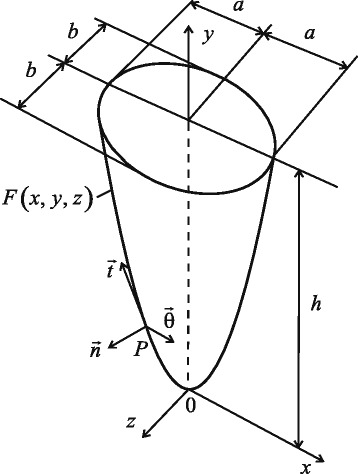
Geometrical shape of tooth root: $\vec {n}$ is normal, $\vec {t}$ is generatrix, $\vec {\theta }$ is tangential to surface of tooth root in point *P*

Following Kawarizadeh et al. (2003), Rees and Jacobsen (1997), it is supposed that the PDL has a Poisson’s ratio equal to 0.49, i.e. effectively incompressible. This means that it should exhibit a fluid-like behaviour, flowing around the surface of the root of the tooth when the latter is displaced to the wall of the dental alveolar bone (Kawarizadeh et al. 2003). Hence, strains and relative shears associated with a normal vector, a generatrix of, and a tangent to, the external surface of the tooth root could be represented in the coordinate system as follows (Provatidis 2001; Van Schepdael et al. 2013):

(3) $${} {\fontsize{9.4pt}{9.6pt}\selectfont{\begin{aligned} \varepsilon_{nn} = - \frac{u_{n}}{\delta}, \varepsilon_{tt} = \varepsilon_{\theta \theta} = 0, \gamma_{n \theta} = - \frac{u_{\theta}}{\delta}, \gamma_{nt} = - \frac{u_{t}}{\delta}, \gamma_{t \theta} = 0, \end{aligned}}}  $$

where *u*_*n*_, *u*_*θ*_ and *u*_*t*_ are displacements of the PDL points, with subscripts *n*, *θ* and *t*, denoting the normal, tangential directions with regard to the root surface, and the generatrix of it, and *δ* being the thickness of the PDL in the normal direction. The normal vector $\vec {n}$, tangential $\vec {\theta }$ to the root surface of the tooth and generatrix $\vec {t}$, as well as its geometrical dimensions are shown in Fig. 1.

Any displacements of the rigid tooth root can be presented as a combination of translational displacements *u*_0*x*_, *u*_0*y*_, *u*_0*z*_ and angles of rotation *θ*_*x*_, *θ*_*y*_, *θ*_*z*_ with regard to the axes of coordinates. Since the thickness of PDL is small, the rotation angles are small, too. Hence, the following linearized expressions can be used:

(4) $$\begin{array}{*{20}l} \begin{aligned} u_{x} &= u_{0x} + z \theta_{y} - y \theta_{z}, u_{y} = u_{0y} - z \theta_{x} + x \theta_{z}, \\ u_{z} &= u_{0z} + y \theta_{x} - x \theta_{y}. \end{aligned} \end{array} $$

After transformation in accordance with Van Schepdael et al. (2013), the relationships between the displacements *u*_*x*_, *u*_*y*_ and *u*_*z*_ of the tooth root and strains in the PDL *ε*_*xx*_, *ε*_*yy*_, *ε*_*zz*_, *γ*_*xy*_, *γ*_*yz*_ and *γ*_*xz*_ can be obtained in Cartesian coordinates.

### Constitutive equations

An overview of specific applications of different models of PDL is given in (Fill et al. 2012). The main drawback of schemes presenting a PDL in simulations as a material with a complex mechanical behaviour is a lack of accurate quantitative data for respective mechanical parameters. For viscoelastic models it is compensated by availability of known magnitudes of relaxation times and elasticity moduli (Komatsu 2010; Qian et al. 2009; Wood et al. 2011), and experimentally determined viscoelastic properties (Bergomi et al. 2011; Ferrari et al. 2008; Natali 2003; Naveh et al. 2012; Toms and Eberhardt 2003; Toms et al. 2002; Yoshida et al. 2001).

Several models of viscoelastic behaviour of the PDL, based on approaches by Maxwell, Voigt and, Kelvin-Voigt, were proposed (Fill et al. 2012). Such material are said to exhibit a *rheological behaviour*. Rheology as a branch of science is concerned with extending continuum mechanics to characterization of flow of materials, with a combination of elastic, viscous and plastic properties by merging elasticity and (Newtonian) fluid mechanics. In particular, materials studied within the framework of rheology could have a memory (so called *hereditary materials*). To model this effect, a fractional calculus can be used, e.g., (Koeller 2010; Uchaikin 2013; West et al. 2003); the history of fractional modelling in rheology is presented in (Rogosin and Mainardi 2014) (see also (Mainardi 2010) and references therein). A fractional viscoelastic model provides a rather natural approach for a study of periodontal membranes. In addition, fractional models (i.e. models with fractional derivatives) are successfully used to solve different problems of mechanics (Rossikhin and Shitikova 2013b; Rossikhin et al. 2014).

A general theory of mechanics of hereditary materials was suggested by Rabotnov (1980) using integral equations; Koeller (1984) reviewed the application of integral equations to viscoelasticity and introduced fractional calculus into the Rabotnov’s theory employing a structural spring-dashpot model, used to generalize the classical mechanical models. Rossikhin and Shitikova (2015) summarized the Rabotnov’s theory (see also (Rossikhin and Shitikova 2014)). The Rabotnov’s fractional exponential function is related to a well-known Mittag-Leffler function (Gorenflo et al. 2014). Using this relation, it can be shown that the Rabotnov’s theory is equivalent to the scheme by Torvik and Bagley based on a fractional polynomial constitutive equation. Thus, a viscoelastic model with a fractional exponential kernel is highly suitable for modelling of mechanical behaviour of biological materials with time-dependent properties. In a viscoelastic scheme similar to the Rabotnov’s model, components of a stress tensor can be presented in the following form, taking into account viscoelastic properties of the PDL:

(5) $${} {\small\begin{aligned} \sigma_{ij} &=\frac{E_{\infty}}{(1\,-\,2\nu)(1\,+\,\nu)} \left\{\!(1\,-\,2 \nu) \varepsilon_{ij}\! -\! \nu_{\varepsilon} {\int\limits_{0}^{t}}{\mathcal{E}}_{\gamma}\! \left(\!-\frac{\tau}{\tau_{\varepsilon}}\right) \varepsilon_{ij} (t\,-\, \tau) d \tau + \right.\\ &\quad\left.+\nu \left(\sum\limits_{k=1}^{3} \varepsilon_{kk} - \nu_{\varepsilon} {\int\limits_{0}^{t}} {\mathcal{E}}_{\gamma} \left(-\frac{\tau}{\tau_{\varepsilon}}\right) \sum\limits_{k=1}^{3} \varepsilon_{kk} (t-\tau) d \tau \right) \right\}, \end{aligned}}  $$

where *τ*_*s*_ is the relaxation time; $\nu _{\varepsilon } = \frac {E_{\infty }-E_{0}}{E_{\infty }}$, *E*_0_ and *E*_*∞*_ are, respectively, the instantaneous (glassy) and relaxed (rubbery) elastic moduli (Rossikhin and Shitikova 2015); and ${\mathcal {E}}_{\gamma } \biggl (-\frac {\tau }{\tau _{\varepsilon }}\biggr)$ is the Rabotnov’s fractional exponential function, which describes the relaxation of volume and shear stresses. It was introduced by Rabotnov in the following form (Rabotnov 1948; 1980):

$${\mathcal{E}}_{\gamma}\left(-\frac{t}{\tau_{\varepsilon}}\right)=\frac{t^{\gamma-1}}{\tau_{\varepsilon}^{\gamma}} \sum\limits_{n=0}^{\infty} (-1)^{n}\frac{(t/\tau_{\varepsilon})^{\gamma n}}{\Gamma \left[ \gamma (n+1)\right]}, $$

where 0<*γ*<1 is the fractional parameter. Note that the Rabotnov’s function is a special case of the classical Mittag-Leffler function widely used in fractional models (see (Gorenflo et al. 2014; Mainardi 2010)).

### Equations of motion

To find the translational displacements and rotation angles in the PDL, the following conditions of the dynamic equilibrium of the tooth root are used:

(6) $$ \begin{aligned} &\iint\limits_{F} (\vec{n} \cdot \sigma) dF + M \frac{d^{2} \vec{u_{0}}}{dt^{2}}-\vec{f}=0,\\ &\iint\limits_{F} \vec{r} \times (\vec{n} \cdot \sigma) dF + J \frac{d^{2}\vec{\theta}}{dt^{2}}-\vec{m}=0, \end{aligned}  $$

where $\vec {m} = (m_{x},m_{y},m_{z})$ is the principal moment of external forces; $\vec {f} = \left (\,f_{x},f_{y},f_{z}\right)$ is the principal vector of external forces; $\vec {r}$ is the radius-vector; $\vec {n}=(n_{x},n_{y},n_{z})$ is the unit normal vector to the surface (1), *σ* is the stress tensor; *M* and *J* are the mass and axial moment of inertia of the tooth root, respectively; $\vec {u}_{0} = (u_{0x}, u_{0y}, u_{0z})$ is the vector of translational displacements of the tooth root along the axes of coordinates, and $\vec {\theta } = (\theta _{x}, \theta _{y}, \theta _{z})$ is the vector of rotation angles of the tooth root with respect to the axes. The components of the displacement vector $\vec {u}_{0}$ and the vector of rotation angles $\vec {\theta }$ are functions of time.

Taking into account relations (2) and (5) one can reduce equations of motion (6) (after some transformations) to the following form:

(7) $$ {}{\fontsize{7.3pt}{9.6pt}\selectfont{\begin{aligned} a_{11}\! \left(\!u_{0x} \,-\,\nu_{\varepsilon}\! {\int\limits_{0}^{t}}\!{\mathcal{E}\!}_{\gamma} \!u_{0x}(t\,-\,\tau) d\tau\!\right) \!+ a_{16}\! \left(\!\theta_{z}\! -\!\nu_{\varepsilon}\! {\int\limits_{0}^{t}}\! {\mathcal{E}}_{\gamma}\theta_{z}(t\,-\,\tau) d\tau\!\right)\! + M \frac{d^{2} u_{0x}}{dt^{2}} \!= f_{x}, \\ a_{22}\left(u_{0y} - \nu_{\varepsilon} {\int\limits_{0}^{t}}{\mathcal{E}}_{\gamma} u_{0y}(t-\tau) d\tau\!\right) + M \frac{d^{2}u_{0y}}{dt^{2}} = f_{y},\\ a_{33}\! \!\left(\!u_{0z}\! - \!\nu_{\varepsilon}\! {\int\limits_{0}^{t}}\!{\mathcal{E}}_{\gamma} u_{0z}(t\,-\,\tau) d\tau\!\right) \!+ a_{34}\! \left(\!\theta_{x}\! -\!\nu_{\varepsilon} {\int\limits_{0}^{t}} \!{\mathcal{E}}_{\gamma} \theta_{x}(t\,-\,\tau) d\tau\!\right)\! + M \frac{d^{2} u_{0z}}{dt^{2}} = f_{z}, \\ a_{43} \left(u_{0z} - \nu_{\varepsilon} {\int\limits_{0}^{t}}{\mathcal{E}}_{\gamma} u_{0z}(t-\tau) d\tau\right) + a_{44} \left(\theta_{x} -\nu_{\varepsilon} {\int\limits_{0}^{t}} {\mathcal{E}}_{\gamma} \theta_{x}(t-\tau) d\tau\right) +\\ +~J_{x} \frac{d^{2} \theta_{x}}{dt^{2}} = y_{f}f_{z} - z_{f} f_{y}, \\ a_{55} \left(\theta_{y} - \nu_{\varepsilon}{\int\limits_{0}^{t}} {\mathcal{E}}_{\gamma} \theta_{y}(t-\tau) d\tau\right) +J_{y}\frac{d^{2} \theta_{y}}{dt^{2}} = z_{f} f_{x} - x_{f} f_{z}, \\ a_{61} \left(u_{0x} -\nu_{\varepsilon} {\int\limits_{0}^{t}} {\mathcal{E}}_{\gamma} u_{0x}(t-\tau) d\tau\right) + a_{66} \left(\theta_{z} - \nu_{\varepsilon}{\int\limits_{0}^{t}} {\mathcal{E}}_{\gamma} \theta_{z}(t-\tau) d\tau\right) +\\ +~J_{z} \frac{d^{2} \theta_{z}}{dt^{2}} = x_{f} f_{y} - y_{f} f_{x}, \\ {\mathcal{E}}_{\gamma} \equiv {\mathcal{E}}_{\gamma}\left(-\frac{\tau}{\tau_{\varepsilon}}\right), \end{aligned}}}  $$

where *x*_*f*_, *y*_*f*_ and *z*_*f*_ are the coordinates of the point where the load is applied. The coefficients of the system (7) are presented in Appendix. These coefficients are calculated for the tooth root with geometrical dimensions *h*=13.0 mm, *b*=3.9 mm and *e*=0.6. Elastic properties of the PDL are assigned by constants *E*_*∞*_=680 kPa and *ν*=0.49 (Tanne et al. 1991). Thickness *δ* of the PDL is 0.229 mm (Provatidis 2001). In this case, *a*_16_=*a*_61_=−44.168 kN and *a*_34_=*a*_43_=59.060 kN. Magnitudes of other coefficients of system (7) are given in Table 1.

**Table 1:** Coefficients of system (7)

*a*_11_ [M N/m]	*a*_22_ [M N/m]	*a*_33_ [M N/m]	*a*_44_ [N ·m]	*a*_55_ [N ·m]	*a*_66_ [N ·m]
5.043	1.090	6.997	578.9	6.137	445.6

The coefficients *a*_*ij*_ depend on the geometrical shape of the tooth root, the Poisson’s ratio as well as the instantaneous and relaxed elastic moduli of the periodontal tissue and are time-independent. Therefore, they could be eliminated from the integrals in Eq. (7).

### Translational displacements of tooth root

During the motion of the tooth root along the y-axis, corresponding to extrusion (or intrusion), the translational displacements along the *x*- and *z*-axes, as well as the angles of rotation vanish, i.e., *u*_0*x*_=*u*_0*z*_=0 and *θ*_*x*_=*θ*_*y*_=*θ*_*z*_=0; the load acts only along the *y*-axis. In this case, one obtains from (7)

(8) $$ a_{22} \left(u_{0y} - \nu_{\varepsilon} {\int\limits_{0}^{t}} {\mathcal{E}}_{\gamma} u_{0y}(t-\tau) d\tau\right) + M \frac{d^{2} u_{0y}}{dt^{2}} = f_{y}.  $$

In the case of translational displacement of the tooth root in a horizontal plane, in particular, along the *x*-axis, *u*_0*y*_=*u*_0*z*_=0 and *θ*_*x*_=*θ*_*y*_=*θ*_*z*_=0. The load acts along the *x*-axis, and its line of action passes through the centre of resistance of the tooth root with coordinates (0,*y*_1_,0). As a result, we have

(9) $$ \begin{aligned} a_{11} \left(u_{0x} - \nu_{\varepsilon} {\int\limits_{0}^{t}}{\mathcal{E}}_{\gamma} u_{0x}(t-\tau) d\tau\right) + M \frac{d^{2}u_{0x}}{dt^{2}} = f_{x}, \\ a_{61} \left(u_{0x} - \nu_{\varepsilon} {\int\limits_{0}^{t}} {\mathcal{E}}_{\gamma} u_{0x}(t-\tau) d\tau\right) = - y_{1}\,f_{x}. \end{aligned}  $$

To obtain the system of equations describing the translational motion of the tooth root along the *z*-axis, it is necessary to equalize displacements *u*_0*x*_ and *u*_0*y*_ and all angles of rotation in (9) to zero. In this case, only the *z*-component of the load acts on the tooth, and its line of action passes through the centre of resistance with coordinates (0,*y*_2_,0).

## Results

### Strains in PDL during translational displacement of tooth root

Physical parameters of the viscoelastic model can be assessed using Eq. 8, since stiffness *a*_22_ of the PDL along the *y*-axis direction is smaller than *a*_11_ and *a*_33_. Duration of the load action on the tooth root is assumed to be large enough (from 0 to 300 s, (Qian et al. 2009; Slomka et al. 2008)), and the mass of the tooth root small (*m*=1·10^−3^ kg). Hence, the inertial term in Eq. (8) can be neglected:

(10) $$ u_{0y} - \nu_{\varepsilon} {\int\limits_{0}^{t}} {\mathcal{E}}_{\gamma} u_{0y}(t-\tau) d\tau = \frac{f_{y}}{a_{22}}.  $$

According to (Rossikhin 2010) solution of this equation can be written as

(11) $$ u_{0y}(t) = \frac{f_{y}}{a_{22}} \left(1+\nu_{\sigma} \frac{t^{\gamma}}{\tau_{\sigma}} \sum\limits_{n=0}^{\infty} \frac{(-1)^{n} (\frac{t}{\tau_{\sigma}})^{\gamma n}}{\Gamma[\gamma (n+1)]} \right),  $$

where $\nu _{\sigma } = \frac {E_{\infty } - E_{0}}{E_{0}}$, *τ*_*σ*_ is the retardation time. Solution (11) corresponds to the initial conditions $u_{0y}(t){|}_{t=0}=\frac {f_{y}}{a_{22}}$ and $\frac {d u_{0y}(t)}{dt}\left |{~}_{t=0}=\frac {d^{2} u_{0y}(t)}{dt^{2}}\right |{~}_{t=0}=0$.

In Eq. (11), stiffness *a*_22_ is known (see Table 1), while the load *f*_*y*_ must be specified. The retardation time *τ*_*σ*_, parameter *ν*_*σ*_ and fractional parameter *γ* are unknown. The magnitudes of these parameters are assessed using the models for the tooth movement with time in the viscoelastic PDL that were analysed in (Qian et al. 2009; Slomka et al. 2008). The tooth displacement with time in the viscoelastic PDL was determined for a continuous load that changed from 0 to 15 N (Qian et al. 2009) as well as for a discrete change in the load magnitude from 0.5 N to 3.0 N with a step of 0.5 N (Slomka et al. 2008); the time intervals were 300 s (Qian et al. 2009) and 1200 s (Slomka et al. 2008). In our case, the calculation of displacements was performed for the time interval from 0 to 300 s; the transition phase was 20–25 s (Qian et al. 2009; Slomka et al. 2008).

**Fig. 2:**
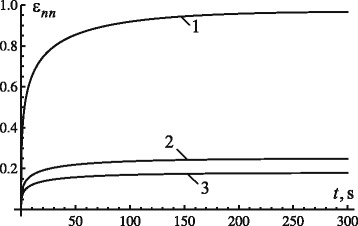
Evolution of strain *ε*_*nn*_ in *xy*-plane during translational displacement of tooth root along *y*-axis: 1 – *x*=0, *y*=0; 2 – $x=\frac {b}{\sqrt {2(1-e^{2})}}$, *y*=*h*/2; 3 – $x=\frac {b}{\sqrt {1-e^{2}}}$, *y*=*h*

For a case of vertical loading of the tooth root, the highest strain in the PDL in the coordinate system (*n*,*t*,*θ*) was *ε*_*nn*_ along the *y*-axis. Evolution of strains *ε*_*nn*_ in the *xy*-plane for different points of the PDL on the surface of the tooth root is shown in Fig. 2. The tooth crown was loaded by a constant compressive force of – 2 N, the fractional parameter *γ* was equal to 0.35; the retardation time *τ*_*γ*_ and the parameter *ν*_*σ*_ were equal to 550 s and 1.3·10^3^, respectively. The values of the above parameters were determined from the condition *ε*_*nn*_≤1 (in accordance with the first expression of relations (3)) for PDL’s points located in the apex of the tooth root.

In addition to strain *ε*_*nn*_, another nonzero strain is *ε*_*nt*_. For the above magnitudes of load, geometric and physical parameters of the tooth root and the PDL as well as those of the fractional kernel, the absolute value of the strain does not exceed 0.45 for the first 300 s.

**Fig. 3:**
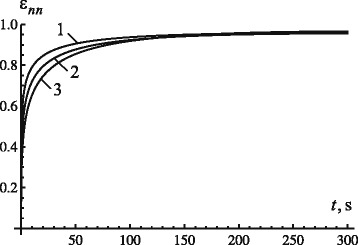
Effect of model parameters on displacement evolution: 1 −*γ*=0.25 and *ν*_*σ*_=1850, 2 −*γ*=0.30 and *ν*_*σ*_=1520, 3−*γ*=0.35 and *ν*_*σ*_=1300

The change of parameter *ν*_*σ*_ for different levels of the fractional parameter provided the same maximum displacements of the tooth root in the PDL. Figure 3 shows the change of displacements with time for the load of 2 N and retardation time of 550 s. The choice of a combination of the constants *γ* and *ν*_*σ*_ was based on the above condition *ε*_*nn*_≤1, following from the first expression in (3).

Figures 2 and 3 demonstrate that a simultaneous change of the fractional parameter *γ* and parameter *ν*_*σ*_ allows us to specify a necessary transitional phase and the maximum displacement of the tooth root in the PDL; this can be achieved for any load. The magnitude of the maximum strain can be defined by changing the magnitude of parameter *ν*_*σ*_, depending on the level of load.

According to results in Fig. 3, an increase of the fractional parameter leads to an increase in duration of the transition phase and in the level of the maximum displacement of the tooth root (for constant values of *ν*_*σ*_ and *τ*_*σ*_).

**Fig. 4:**
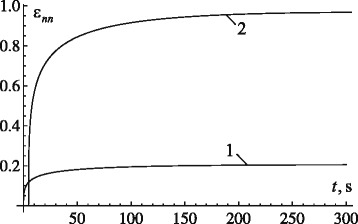
Evolution of strain *ε*_*nn*_ in *xy*-plane during translational displacement of tooth root along *x*-axis for various levels of load *F*: 1 – 2 N; 2 – 9.5 N

Evolution of the normal strains *ε*_*nn*_ for the second particular case, corresponding to Eq. (9), are shown in Fig. 4 (the magnitudes of dimensions of the tooth root, constants of elasticity of the periodontal ligament remained the same). Since in this case the largest deformations occur near the alveolar crest, the deformations at the point with coordinates $\left (x=\frac {b}{\sqrt {1-e^{2}}},0,h\right)$ are defined. Parameters *γ*, *τ*_*γ*_ and *ν*_*γ*_ of the relaxation kernel are equal to 0.35, 550 s and 1.3·10^3^, respectively.

Figure 4 demonstrates that normal strains during the translational motions of the tooth root along the x-axis under the load of 2 N do not exceed 0.2. This can be explained by higher stiffness *a*_11_ of the PDL compared with *a*_22_. To displace the tooth root by distance *δ* along the normal to the surface in *xy*-plane, it is necessary to apply a force of some 9.5 N (Fig. 4).

### Hydrostatic stress of PDL

As known, regions of the PDL exposed to highest hydrostatic stresses govern a bone-remodelling process during an orthodontic tooth movement (De Pauw et al. 2003; Middleton et al. 1996; Vollmer et al. 1999). Hydrostatic stress is determined as

(12) $$ \sigma_{h} = \frac{1}{3} (\sigma_{xx}+\sigma_{yy}+\sigma_{zz}).  $$

As follows from (12) and the discussion above, the hydrostatic stress in the PDL during the translational displacement of the tooth root along the vertical axis is

$$\sigma_{h} = \frac{E u_{0y} \cos(\alpha)}{3\delta(1-2\nu)}. $$

**Fig. 5:**
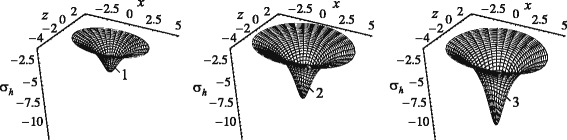
Diagrams of hydrostatic stresses at different moments during translational displacement of tooth root along *y*-axis: 1 – 1 s; 2 – 10 s; 3 – 300 s

Diagrams of distribution of hydrostatic stresses on the tooth-root surface at various times are presented in Fig. 5 for the same magnitudes of load, geometric and physical parameters of the tooth root and the PDL.

Apparently (see Fig. 5), only areas in the close vicinity of the root’s apex are characterised by hydrostatic stresses of considerable magnitudes. The highest stresses for the vertical displacement of the tooth root occur at its apex, while the lowest are observed near the alveolar crest. At *t*=1 s, the hydrostatic stress in the apical region is larger than that near the alveolar crest by approximately 14.1 times. With continuing load action, this ratio increases: at *t*=10 s it is 14.25, at *t*=300 s it is 14.4. A prolonged action of high hydrostatic stresses in the apical region of the tooth root can lead to bone resorption and detrimentally affect the patient. Note that bone resorption in the apical region during the tooth motion (including intrusion) was described in (Jeon et al. 1999; Mohandesan et al. 2007).

**Fig. 6:**
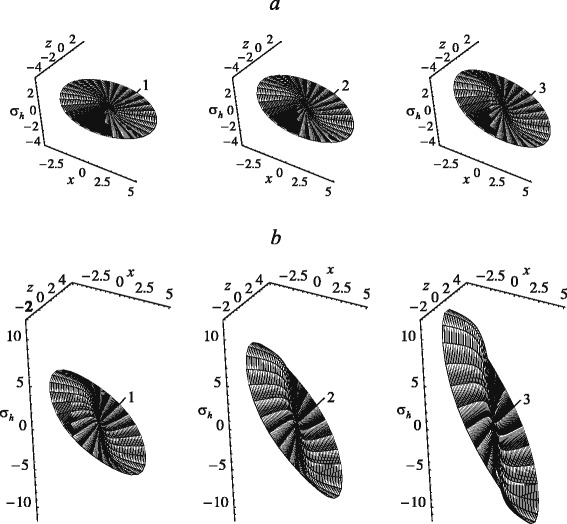
Evolution of hydrostatic stresses with time during translational displacement of tooth root along *x*-axis under 2 N (**a**) and 9.5 N (**b**): 1 – 1 s; 2 – 10 s; 3 – 300 s

Stress distributions on the internal surface (1) of the PDL during the translational displacement of the tooth root along the *x*-axis under loads of 2 N and 9.5 N are shown in Fig. 6. The dimensions of the tooth root, elastic parameters of the PDL are the same, parameters *γ*, *τ*_*γ*_ and *ν*_*γ*_ of the relaxation kernel are equal to 0.35, 550 s and 1.3·10^3^, respectively. In Fig. 6, the coordinates *x* and *z* are in millimeters while the hydrostatic stress is in MPa.

As seen in Fig. 6, the hydrostatic stress on a part of the internal surface of the PDL corresponding to *x*>0 are compressive; on the opposite side of the PDL the tension occurs. This indicates the bone-resorption process in the load direction and bone remodelling on the opposite side of the tooth root. The largest stresses are observed near the alveolar crest. At the point of the PDL corresponding to the apex of the tooth root the hydrostatic stresses vanish. However, in the apical region of the PDL, in particular at *x*>0.9 mm, the stresses reach sufficiently large values, comparable to those near the alveolar crest. Therefore, we can conclude that the outer contours of the hydrostatic-stress diagrams are limited by nearly straight portions (except for a small apical region). The bone remodelling would occur uniformly along the root surface during the translational displacement along the *x*-axis, while beneath the apex of the tooth root the bone would not change. An increase in the maximum hydrostatic stresses in the PDL with time during translational displacements of the tooth along the *y*-axis and *x*-axis occur in a similar way. The magnitude of maximum stress at *t*=10 s and *t*=300 s exceeds the respective value at *t*=1 s by a factor of approximately 1.75 and 2.55, respectively.

### Effect of series truncation

A number of terms in the series of the approximate solution (11) affects substantially the calculated displacements of the tooth root with time. Especially significant is their impact at small values of the fractional parameter. In particular, when *γ*=0.25, the effect of the number of terms of a series becomes negligible for *n*≥20, for *γ*=0.5 this is achieved at *n*≥10, and for *γ*=0.75 at *n*≥3.

### Effect of inertia

To assess the effect of inertia, presented by the term $M \frac {d^{2} u_{0y}(t)}{dt^{2}}$ in solution (11), calculations were performed for the following ranges of parameters: retardation time – between 350 s and 550 s, the fractional parameter – from 0.25 to 0.90, and parameter *ν*_*σ*_ – from 1.3·10^3^ to 1.8·10^3^. The tooth mass, as discussed, was 1·10^3^ kg, while the tooth-root dimensions and the elastic properties of the PDL were as above. The analysis indicated that in the time interval from 0 to 300 s the contribution of the inertial term was of the order of 10^−11^ m/s^2^ to 10^−10^ m/s^2^. Thus, the solution in the form of (11) can be used as a sufficiently good approximation of the vertical movement of the tooth root.

## Discussion

The aim of this study is the development of a mathematical model for description of experimentally observed viscoelastic and time-dependent behaviours of the PDL. In particular, the analysis is focused on the evolution of translational displacements of the tooth root in the PDL under the vertical load (intrusion). The calculated tooth- root displacement with time at a constant load allowed comparing the behaviour of the viscoelastic model with the fractional exponential kernel with that of the known nonlinear viscoelastic model of the tooth-root movement developed in the studies (Qian et al. 2009; Slomka et al. 2008). The model was used to determine the level of hydrostatic stresses in the PDL under the constant intrusive load. The analysis showed that these stresses in the PDL remained practically constant along the surface of the tooth root, except for the region near the root apex. Hydrostatic stresses in this region were significantly higher, indicating potential bone resorption during the orthodontic motion.

## Conclusions

The considered model employs the relaxation kernel with a fractional exponential function and is an extension of the linear scheme for an almost incompressible PDL (with the Poisson’s ratio equal to 0.49), presented in studies (Provatidis 2001; Van Schepdael et al. 2013) it describes both the elastic and viscoelastic behaviours of the PDL. The current lack of experimental data on the time-dependent behaviour of the PDL under various loading conditions hinders development of adequate analytical approaches. One of the limitations of the suggested approach is the increase in the maximum displacement of the tooth with the increased load (similar to the behaviour of the model (Slomka et al. 2008)). At the same time, the proposed model allows generalization of the known analytical models of the viscoelastic PDL by introduction of the instantaneous and relaxed elastic moduli, as well as the fractional parameter. The advantage of this model is in the use of the fractional parameter *γ* and the parameter *v*_*σ*_ improving the description of various pathological processes and age-related changes in the PDL. The fractional parameter makes it possible to take into account different behaviours of the periodontal tissue under short- and long-term loads. For instance, it allows assessing the change in the time interval of a transition phase for a given maximum displacement. Another advantage of the phenomenological model proposed in this study is its capability to predict the behaviour of the PDL in conditions, not feasible in the experiment.

The developed approach can be applied to determine a magnitude of a load for orthodontic tooth movement corresponding to optimal stresses, as well as to simulate bone remodelling on the basis of changes of stresses and strains in the PDL during orthodontic movements.

## Appendix

The coefficients of system (7) has the following form:

$$\begin{array}{@{}rcl@{}} a_{11} = A \iint\limits_{F} (b^{2} (1-2\nu) \cos(\alpha) - 2 h(2 H x(1-e^{2}) (1-\nu)+G z (1-2\nu)) \sin(\alpha))d F, \\ a_{16}=a_{61} = - A \iint\limits_{F} ((4 (1-e^{2}) h \nu x^{2} + b^{2} y(1-2\nu)) \cos(\alpha)+ \\ +\,(b^{2} H x (1-2\nu) +2 h y (2 H x (1-e^{2})(1-\nu) +G z (1-2\nu))) \sin(\alpha))d F, \\ a_{22} = A \iint\limits_{F} (b^{2} (1-\nu) \cos(\alpha) +h(1-2\nu) (H x (1-e^{2})+G z) \sin(\alpha))d F, \\ a_{33} = A \iint\limits_{F} (b^{2} (1-2 \nu) \cos(\alpha)+2 h (H x (1-e^{2}) (1-2 \nu) + 2 G z (1-\nu) \sin(\alpha))d F,\\ a_{34} = A \iint\limits_{F} ((b^{2} y (1-2\nu)+4 h \nu z^{2}) \cos(\alpha)+\\ +\,(2 H h x y (1-e^{2}) (1-2\nu)+G z (b^{2} (1-2\nu)+4 h y (1-\nu))) \sin(\alpha))d F,\\ a_{44} = A \iint\limits_{F} ((2 h y z^{2} + b^{2} ((1-2\nu) y^{2} +2(1-\nu)z^{2}))\cos(\alpha) + \\ +\, (2 h H x (1-e^{2})(1-2\nu)(y^{2}+z^{2}) + G z (b^{2} y +2 h (2 y^{2} (1-\nu) + (1-2\nu) z^{2}))) \sin(\alpha))d F, \\ a_{55} = A \iint\limits_{F} (b^{2} (1-2\nu) (x^{2}+z^{2})\cos(\alpha)+\\ +\,2 h(G z(e^{2} x^{2} + (1-2\nu)(x^{2}+z^{2})) + H x ((x^{2}+z^{2})(1-2\nu) -e^{2} ((1-2\nu) x^{2} + 2(1-\nu)z^{2}))) \sin(\alpha))d F, \\ a_{66} = A \iint\limits_{F} ((2 h x^{2} y (1-e^{2}) +b^{2}(2(1-\nu)x^{2} + (1-2\nu)y^{2}))\cos(\alpha) + \\ +\, (b^{2} H x y + 2 h(H x (1-e^{2})((1-2\nu)x^{2}+2 (1-\nu)y^{2}) +G(1-2\nu)(x^{2}+y^{2})z))\sin(\alpha))d F, \\ A = \frac{E_{\infty}}{2 \delta b^{2} (1+\nu) (1-2 \nu)}, H = \frac{x (1-e^{2})}{\sqrt{(1-e^{2})^{2}x^{2}+z^{2}}}, G = \frac{z}{\sqrt{(1-e^{2})^{2}x^{2}+z^{2}}}. \end{array} $$
